# "*They just come*, *pick and go*." The Acceptability of Integrated Medication Adherence Clubs for HIV and Non Communicable Disease (NCD) Patients in Kibera, Kenya

**DOI:** 10.1371/journal.pone.0164634

**Published:** 2016-10-20

**Authors:** Emilie Venables, Jeffrey K. Edwards, Saar Baert, William Etienne, Kelly Khabala, Helen Bygrave

**Affiliations:** 1 Médecins Sans Frontières Southern Africa Medical Unit, Cape Town, South Africa; 2 Division of Social and Behavioural Sciences, School of Public Health and Family Medicine, University of Cape Town, Cape Town, South Africa; 3 Medical Department, Operational Centre Brussels, Médecins Sans Frontières Belgium, Brussels, Belgium; 4 Médecins Sans Frontières, Nairobi, Kenya; University of Pittsburgh, UNITED STATES

## Abstract

**Introduction:**

The number of people on antiretroviral therapy (ART) for the long-term management of HIV in low- and middle-income countries (LMICs) is continuing to increase, along with the prevalence of Non-Communicable Diseases (NCDs). The need to provide large volumes of HIV patients with ART has led to significant adaptations in how medication is delivered, but access to NCD care remains limited in many contexts. Medication Adherence Clubs (MACs) were established in Kibera, Kenya to address the large numbers of patients requiring chronic HIV and/or NCD care. Stable NCD and HIV patients can now collect their chronic medication every three months through a club, rather than through individual clinic appointments.

**Methodology:**

We conducted a qualitative research study to assess patient and health-care worker perceptions and experiences of MACs in the urban informal settlement of Kibera, Kenya. A total of 106 patients (with HIV and/or other NCDs) and health-care workers were purposively sampled and included in the study. Ten focus groups and 19 in-depth interviews were conducted and 15 sessions of participant observation were carried out at the clinic where the MACs took place. Thematic data analysis was conducted using NVivo software, and coding focussed on people’s experiences of MACs, the challenges they faced and their perceptions about models of care for chronic conditions.

**Results:**

MACs were considered acceptable to patients and health-care workers because they saved time, prevented unnecessary queues in the clinic and provided people with health education and group support whilst they collected their medication. Some patients and health-care workers felt that MACs reduced stigma for HIV positive patients by treating HIV as any other chronic condition. Staff and patients reported challenges recruiting patients into MACs, including patients not fully understanding the eligibility criteria for the clubs. There were also some practical challenges during the implementation of the clubs, but MACs have shown that it is possible to learn from ART provision and enable stable HIV and NCD patients to collect chronic medication together in a group.

**Conclusions:**

Extending models of care previously only offered to HIV-positive cohorts to NCD patients can help to de-stigmatise HIV, allow for the efficient clinical management of co-morbidities and enable patients to benefit from peer support. Through MACs, we have demonstrated that an integrated approach to providing medication for chronic diseases including HIV can be implemented in resource-poor settings and could thus be rolled out in other similar contexts.

## Introduction

The number of people on antiretroviral therapy (ART) for the long-term management of HIV in low- and middle-income countries (LMICs) is continuing to increase. Ambitious targets set by The United Nations Programme on HIV/AIDS in its 2014 90-90-90 campaign, newly released WHO guidelines on early ART initiation along with recent trial data supporting a move to a test and treat approach, will result in a dramatic increase in the number of people requiring long-term ART within already strained health-care systems [1–4].

Concurrently, there is a rapid epidemiological transition occurring in LMICs, especially Sub-Saharan Africa [5], in which an increasing prevalence of Non-Communicable Diseases (NCDs) such as hypertension and diabetes are being seen, particularly in urban informal settlements [6]. Based upon current projections within Kenya, NCDs will surpass communicable diseases such as HIV as the leading cause of mortality by 2025 [7].

There have, however, been great improvements in access to HIV care and treatment in Sub-Saharan Africa in the last decade which have made a huge impact on people’s lives and how they manage their chronic conditions. People living with HIV who are on ART and maintain virological suppression are aging and now more likely to die of an NCD than other causes [8], yet in addition, HIV and ART are linked with an increased risk of developing hypertension and/or diabetes [9, 10]. In many LMICs, access to NCD care and treatment remains limited due to a lack of treatment guidelines, trained providers at the primary care level and the cost of medications [11, 6]. This problem is magnified within impoverished urban informal settlements such as Kibera, on the outskirts of Nairobi in Kenya [12].

In many LMICs the lack of human resources and infrastructure make health-care difficult for many people to access. Patients on chronic medication for HIV and/or NCDs face challenges including living long distances from health-care facilities, lacking the economic resources to travel, being unable to take time off work to wait in addition to the challenge of facing stigma which makes many people reluctant to access local services or seek support from others [13].

In response to the growing need to provide large volumes of HIV patients with ART, there have been significant adaptations in how medication is delivered so as to enable more people to start and continue treatment. Key enablers of ART scale-up have included extending the time between drug refills and decreasing the frequency of clinical follow-up to reduce the time-burden on stable patients; task-shifting to lower cadres such as nurses and counsellors; decentralisation of services to primary care facilities and implementing facility- and community-based clubs and community ART groups [14–16]. Key to these programmatic adaptations has been enhancing patient self-management and separating clinical consultations from drug refills [17, 18], and there is the potential to learn from and expand these models by applying lessons from the scale up of ART delivery to the provision of NCD care [19].

To address the increasing numbers of patients requiring chronic HIV and/or NCD care in the informal settlement of Kibera in Kenya, Médecins Sans Frontières (MSF) established Medication Adherence Clubs (MACs) in partnership with the Kenyan Ministry of Health. MSF has provided HIV/TB services in primary health-care settings in Kibera since 2003, and integrated NCD management from 2009. A fast-track system providing a three month supply of medication to stable patients directly through the pharmacy was introduced in 2010, and MACs were implemented in 2013.

Kibera is one of the largest informal settlements in Africa, located just outside the centre of Nairobi, Kenya, with a population estimated at between 235,000 and 270,000 people [20]. HIV, hypertension (HT) and diabetes mellitus (DM) prevalence in Kibera is estimated to be 12.6%, 12.3% and 5.6% respectively [21, 22, 12]. In Kibera South Clinic where this study took place, MSF currently supports a cohort of over 5000 HIV patients and 2500 NCD patients who require chronic medication. Stable chronic patients can collect their drugs through standard clinic appointments, fast-track appointments where refills are collected from the pharmacy every three months with an annual clinical consultation, or through a MAC. Clinicians refer patients to MACs or fast-track appointments depending on certain clinical criteria, but patients do not have to join the model of care proposed to them.

MACs provide a medication refill system for HIV, DM and HT patients who meet defined clinical eligibility criteria. Clubs consist of between 10 and 30 members who meet quarterly in the afternoons or weekends in a dedicated clinic space, and sessions last between 30 and 90 minutes. Members are not required to disclose their HIV/NCD status with the rest of the group if they do not want to. MACs are facilitated by two non-medical health educators, who begin each session with a health talk and discussion. During the research period, these talks included discussions on HIV, nutrition, exercise and cervical cancer screening. All patients are weighed and their blood pressure taken during the sessions. Medications for MAC members are pre-packed and labelled by the pharmacy and provided to each patient in a sealed paper bag. Patients are asked to verify that they have the correct medications before leaving the clinic. Before leaving, patients are given the date of their next meeting and given the opportunity to discuss any issues they have.

We discuss the results from a qualitative study assessing the first year of implementation of the MACs in Kibera from the perspective of health-care workers and patients.

## Methods

### Study design

This is a qualitative study, consisting of In-Depth Interviews (IDIs), Focus Group Discussions (FGDs) and participant observation exploring experiences and perspectives on the MAC model of care, and whether integrating HIV and NCD patients in the same group was acceptable in this context.

### Study sites

The study was conducted at Kibera South Clinic, in the urban slum of Kibera in Kenya. All IDIs and FGDs took place in clinical consultation rooms or the dedicated MAC area within the clinic.

### Study period

The research was carried out over a two month period, from January to March 2015. Fieldwork took place during weekday clinic hours as well as on Saturdays when MACs were scheduled.

### Participant recruitment

Participants were purposively selected for the study, based on several eligibility criteria which are outlined in more detail below:

- Adults who are HIV positive, on ART and members of a MAC
- Adults who are HIV positive, on ART and eligible to join a MAC but not currently a MAC member
- Adults who are HT/DM patients and members of a MAC
- Adults who are HT/DM patients and eligible to join a MAC, but not a MAC member
- Health-care workers providing HIV/HT/DM care to MAC and non-MAC patients

MAC and non-MAC members meeting the different criteria outlined above were contacted telephonically and invited to come to the clinic for an IDI or an FGD. In addition, pre-identified participants who met these criteria were approached in the waiting room when they were waiting for their scheduled consultation and asked if they would like to take part in the research study after their appointment. MAC participants were also approached as they waited for their scheduled MAC meetings to begin–they were given information about the study, before being invited to join if they were interested.

Participants were invited specifically for an IDI or an FGD based on their eligibility criteria and their availability. Participants were offered a choice of research activity, so if they did not want to participate in a group discussion but were interested in being interviewed, they could take part in an IDI instead. ‘Fast-track’ patients who were collecting their refills every three months directly from the pharmacy were not included in the study. Health-care workers providing HIV/HT/DM care were eligible to participate in key informant interviews and FGDs, and a purposive sample of nurses, clinicians, nutritionists, counsellors, pharmacists and health educators was selected. This sample was chosen to ensure that people who have different roles in the running of MACs were involved in the study, so as to gain a wide range of perspectives.

A total of 19 IDIs, 10 FGDs and 15 sessions of participant observation within MAC sessions and the clinic were conducted (Table 1). Of the ten FGDs that took place, two were with health-care workers, four with MAC patients and four with non-MAC patients.

**Table 1:** Study participants by mode of data collection.

	Participants in focus group discussions n (%)	Participants in in-depth interviews n (%)
Health-care workers	16 (18)	9 (47)
MAC patients	36 (42)	6 (32)
Non-MAC patients	35 (40)	4(21)
Total	87 (100)	19 (100)

A total of 106 people participated in the study (Table 2).

**Table 2:** Demographic description of research participants.

	Male n (%)	Female n (%)	Total number of participants n (%)
**Health-care workers**	9 (23)	16 (24)	25 (23)
**MAC patients**	19 (49)	23 (34)	42 (40)
**Non-MAC patients**	11 (28)	28 (42)	39 (37)
**Total**	39 (100)	67 (100)	106 (100)

### Data collection

Data was collected by the principal investigator, an anthropologist who was external to the Kibera project, with assistance from a local Swahili-speaking Kenyan research assistant who had experience working in the study site and in qualitative methodologies, translation and transcription. IDIs and FGDs were conducted in English or Swahili depending on the preferred language of the participant(s). The majority of FGDs were conducted in a combination of the two languages and participants would switch between the two as they spoke. Translations were checked for consistency with another native Swahili speaker who was not involved in the data collection. FGD participants were divided into the following groups to allow for discussion between those who had different experiences: health-care workers, HIV and NCD patients who were in MACs; NCD patients who were eligible for MACs but had not joined and HIV patients who were eligible for MACs but had not joined.

Participant observation was conducted by the PI and a local research assistant, who sat in on 15 different MAC sessions (each with different members) in order to observe the discussions, health education sessions and interactions between club members. Observations took place in MACs conducted by different facilitators, as well as on different days of the week. Data from participant observation took the form of handwritten notes.

The following themes were discussed in IDIs and FGDs: experience of collecting chronic medication, awareness of different strategies for ART/NCD drug refills; knowledge of and/or experience of MACs; incentives and barriers to joining a MAC, support strategies and disclosure and suggestions for improving services for chronic patients.

A total of 5028 HIV and NCD patients were receiving chronic medication refills in Kibera South Clinic at the time of the research. Of these, 2212 (44%) were eligible to join a MAC, and of those who were eligible, 1428 (28% of the total cohort) were then enrolled into the clubs. The median age of the MAC patients who participated in the study was 48 years, with 71% of MAC patients being HIV positive and 64% being female.

### Data analysis

Where necessary, data were translated from Swahili into English during transcription. All data were then transcribed and a coding framework was developed based on themes emerging from relevant literature and the FGD, IDI and participant observation data. A thematic analysis approach was used, in which key themes around the acceptability and perceptions of MACs were compared across focus group discussions, interviews and participant observation. All transcripts and fieldnotes were coded through an iterative process that involved firstly coding the text into broad themes, then grouping these together and developing sub-themes. New themes were discussed with the rest of the research team and project staff as they emerged. Emphasis was also placed on comparing the perspectives of HIV positive and NCD patients, as well as MAC-members and non-MAC members, to see if they had differing experiences of MACs. NVivo qualitative data analysis software was used for data analysis.[23]

### Limitations

The study was conducted after one year of implementation of the MACs, therefore some of the patients had only attended one or two MAC meetings when the research took place. All IDIs and FGDs took place in the health-care facility due to concerns about security in the wider Kibera community, which may have impacted upon the openness of the participants, particularly when they were asked to reflect critically upon the health-care services they were being provided with. Whilst the PI was external to the project, it is possible that she was perceived as belonging to the clinic team, meaning patients and health-care workers may not have been comfortable to voice their views in front of someone they perceived as a staff member.

### Ethical considerations

Ethical approvals were granted by Médecins Sans Frontières (ID 1436) and the Kenyan African Medical and Research Foundation (AMREF) ethical review committees (P140/2014) in 2014. Written informed consent (in English or Swahili) was obtained from all participants prior to data collection. Refreshments were provided during IDIs and FGDs, and transport costs were reimbursed for patients who had travelled to the clinic specifically to participate in the study. The demographics of health-care workers have not been included in citations so as to maintain confidentiality.

## Results

### Acceptability: who do MACs work for and why?

Health-care workers and HIV/NCD patients in Kibera found MACs to be an acceptable, time-saving way of providing and receiving chronic medication. MAC members preferred the clubs to clinic appointments because they reduced the number of clinic visits which saved them time and money; gave them information on a range of health issues and provided them with additional support from health-care workers and their peers through the group environment. Health-care workers cited similar advantages for patients, with one male health-care worker describing the benefits of the MACs as being ‘*they come*, *pick and go*!’ and a female health promoter calling them ‘*the group that educates*.’

The patients below discussed how joining a MAC saved them time and made it more convenient for them to collect their medication whilst working:

[A]t work when you asked for permission to come for treatment, you took the whole day in the hospital and maybe you had only requested permission for a half day so when you go back to work they scold you. So with this way of coming here [the MAC], I am able to go to work for a half day, then I ask for permission to come to the clinic to pick my medication. At least the boss will see that I am not being too bothersome all the time.

53 year old HIV positive female MAC member

This 45 year old male MAC member agreed:

[F]or me it was a very good idea because I work so I’m able to get some time off work so I can come here and get my medication and still not lose my day at work.

Whilst the overall acceptability of the MAC model of care was high, IDIs and FGDs with non-MAC patients showed that there was little knowledge of the clubs outside of MAC members and health-care workers. The recruitment process for joining a MAC was clinician-led rather than patient-driven, with very few research participants stating they had enquired about the MACs themselves. Some clinicians reported that they did not always have enough time to discuss the MACs and their eligibility criteria in detail with their patients. Once patients joined a MAC they spoke favourably of its benefits, but did not always understand the eligibility criteria before being recruited. Some patients (MAC members and non-members) were unaware that the groups were made up of people with different chronic diseases, thinking the MACs were only for people who were HIV positive. During FGDs and IDIs, patients talked about ‘*qualifying*’ for the MACs, implying that they were aware of existing eligibility criteria but did not know what enabled someone to ‘*qualify*’.

The main reason non-MAC patients gave for not joining a club (or the fast-track drug refill system) was that they did not know that they were able to: this was due to a lack of service promotion within the clinic as well as health-care workers reporting that they did not have enough time for recruitment during consultations. Health-care workers often described feeling rushed due to the large numbers of consultations, and did not have the time to describe the MACs to everyone who was eligible. Other reasons for not joining MACs included the afternoon or weekend sessions being inconvenient and health-care workers reporting that some patients preferred the fast-track system in which they collected their refills directly from the pharmacy as they did not want to be in a group. Some HIV positive women with HIV positive children did not want to join a MAC because they wanted to have their appointments at the same time as their children.

The majority of health-care workers felt that MACs eased their workload and reduced waiting times, but also felt that they enabled clinicians, such as the one cited below, to spend more time with more complicated patients who needed specialised clinical support:

One of the biggest challenges is the queuing time… Patients who are stable spend almost all day here…just for their prescription and then they go. [Through the MACs] I am giving room to patients who are patients. These guys are stable, yes? I’m not seeing a reason why they need to come to a clinician every three months.

Despite recognition of an overall decrease in workload by the majority of health-care workers because of reduced numbers of patients waiting, pharmacy staff felt that pre-packing drugs for large numbers of patients at the same time increased their work before MAC sessions.

### Integrated MACs for HIV and NCD patients: benefits and challenges

The majority of research participants—patients and health-care workers—were supportive of integrating HIV, DM and HT patients in MACs, although in some of the FGDs it became apparent that not everyone realised that the clubs were for people with different chronic health conditions, in part due to the recruitment challenges described above. Health-care workers found that the combined groups were easier for them to implement and organise, and believed that combining patients together reduced stigma as those living with HIV were not treated differently from people with diabetes or hypertension.

MAC patients found the integrated clubs beneficial as they learned about chronic health conditions other than their own: one diabetic MAC member described how she had learnt the importance of regular HIV testing from someone in the group who was HIV positive.

In relation to stigma, some MAC members talked about HIV being treated like ‘*any other chronic disease’* because there was no distinction made between HIV and other NCDs, and said the integrated groups were not a concern for them and did not impact upon the care or treatment they received. This was also supported by the nurse cited below:

[I]t’s a good thing to mix them up because the idea is that we all have chronic diseases that are lifelong. [T]he medication is lifelong… whether you have HIV or diabetes or hypertension. And we can keep supporting each other. For me, it’s a good thing.

Some non-MAC, HIV positive patients with little knowledge of the clubs were concerned about being required to disclose their HIV status during a MAC and feared that they would have to do so in front of a group of people they might recognise from their community, thus causing them to be stigmatised. Once they understood that they did not have to, they were supportive of the idea of joining a club.

This female health promoter who was involved in the running of MACs discussed stigma and explained why she thought people may not want to join a MAC:

Maybe you know me, I know you, you are my neighbour and you have never known about my status. [S]ometimes it makes it difficult for them to remove their drugs and check in front of other people.

Integrated MACs were acceptable to health-care workers and patients both as a concept and in their practical implementation–whilst there were some concerns about the stigmatisation of HIV positive patients in groups with non-HIV patients, these were not raised by club members themselves but came from those with less knowledge of MACs.

### Peer support in the MACs

Whilst peer support was cited as an important benefit of the MACs by health-care workers, this was not observed during sessions, and few MAC members could give specific examples of times they had given or received support to or from another member when they were asked in IDIs or FGDs. HIV positive and NCD patients in MACs stated that the group enabled them to ‘*talk to each other and encourage each other’* and said the clubs enabled them to ‘*make new friends and get encouragement’* but relationships rarely seemed to extend outside of the clubs, which may also be related to the lack of cohesive community in an urban slum setting. MAC members all accessed the services of Kibera South Clinic, but did not necessarily live in the same area of Kibera or know people from within their communities who were also accessing chronic medication.

One nurse believed MACs created a sense of ‘*belonging*’ amongst patients because of the group dynamic. She considered HIV, DM and HT to all be chronic diseases, without distinguishing between them:

I think it’s a good idea to put them together. It is all a chronic disease. HIV is no longer what it used to be. But when you put them together they find a sense of belonging… I think when you put them together it’s good and it reduces the stigma.

This view was also supported by MAC members such as this one:

I think if we are separated, some might start feeling as though [their] condition was bad or worse compared to someone else’s but when we are mixed, even as I pick my medication there you cannot tell which medication the other person has taken, so I feel that that really helps.

Female HIV positive MAC patient

The integration of patients with chronic conditions was seen as a benefit by the majority of health-care workers who participated in the study, although this clinician found the concept of combined MACs challenging:

The whole idea of having MACs is to empower the patients, number one, and to be self-reliant. In the MACs we should be able to talk about so many things, so if you put a diabetic and an HIV patient and a hypertensive together, the issues that they go through are very different. [I]t’s easier to deal with people that you have something in common with.

## Discussion

The above results show that it is feasible and acceptable to apply ART delivery models to HIV, HT and DM patients in an integrated model of care such as a MAC. Adherence clubs do not have to be limited to people who are HIV positive, particularly considering the global increase in NCD prevalence. If, as suggested by the literature, the number of HIV and NCD patients in LMICs such as Kenya continues to grow, it will become increasingly difficult to offer support if patient-centred drug refill systems such as MACs do not become widespread [5–7]. Such models have already been used across Southern Africa to provide large numbers of stable HIV-patients with treatment and support [15–18], and an integrated model such as the MACs is an innovative way of responding to HIV and NCD patient needs with limited human and infrastructural resources.

As with other club and community models of care such as those in Mozambique and South Africa [15–18], the main advantage of the Kibera MACs for patients and health-care workers is the reduction in waiting time and number of appointments [24]. MACs–like other chronic clubs across Southern Africa—are managed and run by non-clinical facilitators such as health promoters or peer educators who are living with HIV—and could be peer-led in future, enabling clinicians to focus on less stable patients who need additional clinical support. Task shifting enables clinicians to focus on more complicated cases and enables stable chronic patients to reduce the number of clinic visits they need to attend.

### Challenges in building peer support

As this study took place after one year of implementation, the MAC model was still relatively new for health-care workers and patients in Kibera. There were challenges in implementation, which although not impacting on patients’ decisions to join a MAC, affected the daily running of the clubs. Participant observation during MAC sessions showed that much of the group facilitation focussed on health education rather than creating links and a peer dynamic among members, and the short time which patients have spent together meant that strong peer support networks had not had the chance to fully develop at the time of the study.

Strengthening peer support networks within and outside of MAC sessions could be beneficial by creating a strong group dynamic which would further engage and empower NCD and HIV patients in their own health-care. Focusing on the similarities between different chronic conditions rather than separating HIV from other diseases is useful, as both HIV and other NCDs have clear treatment goals and strategies for lifestyle changes and adherence. Group discussions around stigma and disclosure tended to focus on HIV patients, but DM and HT patients also talked about the challenges in disclosing their chronic diseases with others, showing the similarities in coping with the health conditions. Adherence strategies and disclosure could thus become the basis for shared peer support in future MACs and as more patients become enrolled into alternative models of care such as this one.

To further assess peer support and understand discrepancies between our observations and IDI and FGD data, we need to better conceptualise the idea of peer support, which could address whether patients discuss common problems together during or outside of MAC sessions; visit each other at home or stay in contact with members outside of the scheduled sessions as well as knowing where other members are if they miss a session. Support could also take the form of psycho-social or emotional support as well as material or financial assistance in a time of need.

### Empowering patients through offering a choice

In order to ensure a model of chronic care which is acceptable to patients–as well as feasible and acceptable to implementing health-care workers–both must be involved in its conceptualisation and where possible, a range of options created to suit their needs. Exploring recruitment into MACs revealed that health-care workers felt pressurised into enrolling patients, and were recruiting them without fully understanding the benefits or what the eligibility criteria were. Patients did not always have the required information about the models of care available to them to make an empowered, informed choice about how they collect their chronic medication. Health-care workers also need to be encouraged and given the appropriate tools to explain all options available to their patients. Demand creation could also be further stimulated by involving networks of people living with chronic conditions in the conceptualisation and implementation of new models as well as the recruitment of new patients from their communities.

### Providing ongoing integrated care for stable HIV and NCD patients

With this initial qualitative study, implementation appears successful and supports earlier quantitative findings surrounding the model which show reduced individual clinic visits, very few referrals back to standard clinical care and reduced patient waiting times as a result of MACs [24]. MACs, however, remain constrained by the clinic setting and opening hours because of the legal limitations of drug dispensing within communities by lay persons in Kenya. These constraints need to be broken down to allow for improved decentralisation and potential scale-up as seen elsewhere for HIV care, where clubs have expanded into community venues to bring them even closer to people’s homes. Further research is needed to consider the feasibility of expanding MACs to include other common NCDs (epilepsy, asthma and sickle cell) and to provide services for adolescents who are not currently included in the clubs in Kibera. Ensuring that HIV and NCD patients have the same duration of time between their medication refills is also essential (especially in the case of HIV patients with co-morbidities), both for the running of the clubs and to strengthen peer support within MACs. We could also consider expanding ART and NCD refills into community venues, as has been done in other contexts, although this would depend upon the strength of the group dynamic.

## Conclusions

The MAC model of care has shown that it is possible for large numbers of stable HIV and NCD patients in Kibera to collect their chronic medication together whilst reducing patient waiting times, number of clinic visits and clinic patient volumes. Extending models of care and service provision such as clubs which were previously only offered to HIV positive cohorts to include NCD patients may help to de-stigmatise HIV through normalising it as an NCD, and enable the strengthening of peer support networks over time. MACs allow for the efficient management of co-morbidities and enable large numbers of stable patients to collect their chronic medication efficiently, whilst simultaneously enabling patients to benefit from peer support and health education. MACs have been shown to be acceptable to health-care workers and patients, but there are still challenges in recruitment–such as ensuring patients have detailed information about the clubs before deciding whether to join–and in developing peer support networks.

Although MACs were found to be an acceptable model of care for health-care workers and patients in Kibera, they are not the only such model suitable for chronic patients. Any model must place the patient at the centre of their treatment and have clear outcomes that benefit both patients and health care systems. MACs, or any other model of chronic care, should be adapted to the local needs and context, and be developed in consultation with health-care workers, community actors and those living with chronic diseases who will ultimately access–and potentially run—the services. Offering patients–whether HIV positive, hypertensive or diabetic–the option to collect their drugs directly from the pharmacy (considering extended pharmacy hours to allow for greater access) would also save time and suit people who are not comfortable collecting medication in a group setting. As alternative refill strategies for ART are being considered at national level in Kenya and by donors this is an opportune moment to consider how an integrated approach to repeat prescribing for chronic diseases including HIV, can be implemented in other resource poor settings.
